# Correcting Sherrington’s Gait Dysfunction With an Off the Shelf Knee Orthotic

**DOI:** 10.33137/cpoj.v3i1.34528

**Published:** 2020-07-15

**Authors:** Ihsan Balkaya, Eric L Altschuler

**Affiliations:** Department of Physical Medicine and Rehabilitation, Metropolitan Hospital, New York, NY, USA.

**Keywords:** Genu recurvatum, Proprioception, Knee Orthotic, Sherrington

## Abstract

This professional opinion describes the use of an off the shelf knee orthotic to correct the gait and functional mobility of a patient with hemisensory loss including proprioception following a stroke and provides supporting video. Interestingly, this case corrects a human analogue of a functional deficit found experimentally in monkeys in the 19th century by Mott and Sherrington.

Orthotics have been used to correct genu recurvatum in patients with hemiparesis following stroke^1, 2^ but have not typically been used to correct a pure proprioception deficit. In our Physical Medicine and Rehabilitation (PM&R) Prosthetics and Orthotics clinic, we have found that off the shelf orthotics can be beneficial in treating gait dysfunction secondary to sensory deficits. This professional opinion describes, with supporting videos, a case where an off the shelf knee orthotic corrected the gait and function of a patient with hemisensory loss including proprioception following a stroke. Interestingly, this case corrects a human analogue of a functional deficit found experimentally in monkeys in the 19th century by Mott and Sherrington.^3^ Mott and Sherrington showed that a purely sensory lesion in monkeys—sectioning the dorsal root ganglia—caused profound gait dysfunction.^3^ Analogously, we saw a patient with a history of left thalamic stroke who presented with the inability to walk more than five minutes before stopping. He had normal strength (5/5) in the right leg, but absent light touch and proprioception (all joints) and his gait demonstrated significant recurvatum (Video 1). A sports knee brace set in fixed 10 degrees of flexion not only prevented recurvatum, but also immediately normalized the gait (Video 2). The patient can now walk more than a mile without stopping, and his quality of life has returned to what it was before the stroke.

**Video 1**: Severe right knee recurvatum and gait dysfunction without the brace.

**Video 2**: No recurvatum and a normal gait with brace on.

It is of interest to note that when the patient in this case tried taking even a single step with the brace on, but his eyes closed, he immediately started to fall. However, when walking with his eyes open and wearing the brace, he did not need to watch his leg, instead simply had to look ahead to where he was going and was able to walk. This indicates that visual feedback on his position and the direction he intended to walk is likely integral to his motor ability to ambulate in the absence of proprioception.

We have been pleased to find that we can provide significant clinical benefit to persons with Mott and Sherrington’s gait dysfunction using a simple, inexpensive, off the shelf knee orthotic and believe this approach has the potential to be implemented worldwide.

## DECLARATION OF CONFLICTING INTERESTS

The authors have no financial or other conflicts. The patient gave written informed consent for use of the videos.

## AUTHORS BIOGRAPHY

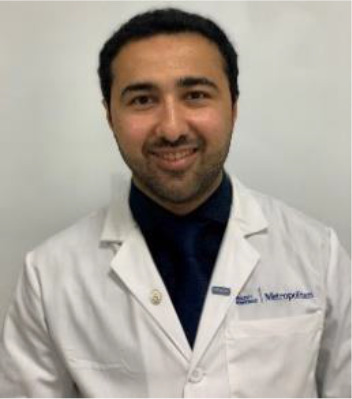



Dr. Ihsan Balkaya is currently a PGY-3 PM&R resident at the New York Medical College/Metropolitan Hospital program. After receiving his medical degree from Istanbul University, he completed his internship at Wayne State University in Detroit, MI. A competitive handball player he is interested in sports medicine.

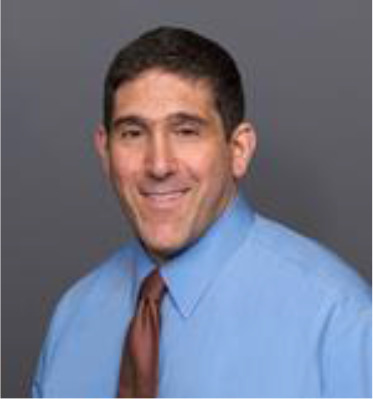



**Eric Altschuler**, MD, PhD is Associate Chief of PM&R at Metropolitan Hospital in New York City and Clinical Associate Professor at New York Medical College. He is board certified in PM&R, Brain Injury Medicine and Neuromuscular Medicine. The main focus of his research is clinically applied cognitive neuroscience.
